# Transaortic septal myectomy at the time of aortic valve replacement for severe aortic stenosis: a case series of 55 cases

**DOI:** 10.1007/s12055-023-01661-x

**Published:** 2023-12-20

**Authors:** Georgios Theodoros Karapanagiotidis, Evangelos Anastasakis, Chrysoula Nana, Philemon Sylvester Gukop, Mustafa Zakkar, Paschalis Tossios, Vasilios Grosomanidis, Despoina Sarridou, Dimitrios Krimiotis, Mazin Abdulla Ibrahim Sarsam

**Affiliations:** 1 Department of Cardiothoracic Surgery, St George’s Hospital, London, UK; 2 AHEPA University Hospital, Thessaloniki, Greece; 3 St George’s University of London, London, UK; 4 Ashford and St Peter’s NHS Foundation Trust, Chertsey, UK; 5 St George’s Hospital, University of London, London, SW17 0QT UK; 6 University Hospital of Leicester NHS Trust, Leicester, UK

**Keywords:** Septal myectomy, Aortic stenosis, Aortic valve replacement

## Abstract

**Introduction:**

Symptomatic aortic valve stenosis (AS) is associated with asymmetric basal septal hypertrophy (ABSH) in 10% of cases. In this cohort, it has been suggested that rectification of the left ventricular outflow tract obstruction (LVOTO) by concomitant septal myectomy (CSM) can improve the results of aortic valve replacement (AVR).

**Objective:**

This study aims to present the technique of AVR with CSM for severe AS with ABSH and to determine the associated early and late post-operative outcomes.

**Methods:**

Fifty-five patients were prospectively recruited to undergo AVR with CSM between 2011 and 2021 at two centres. The primary outcomes were mortality within 30 days, incidence of post-operative ventricular septal defects (VSD) and prosthetic valve sizing. The secondary outcomes were in-hospital complications, permanent pacemaker implantation (PPI), survival at 15 months and changes on transthoracic echocardiogram.

**Results:**

Post-operative mortality was 1.8% and this figure was unchanged at 15-month follow-up. No patients developed a post-operative VSD. Intra-operatively, it was found that in 94.6% cases the direct valve sizing increased by one, when compared to the measurement made before CSM. The indexed effective orifice area (iEOA) was > 85 cm^2^/m^2^ in 96.4% and no patients had an iEOA ≤ 0.75 cm^2^/m^2^. Four patients (7.3%) required PPI due to complete atrioventricular block.

**Conclusion:**

AVR with CSM is a simple technique that can be utilised in severe AS with ABSH. There does not appear to be an increase in mortality or incidence of iatrogenic VSDs. Importantly, CSM allows for the implantation of a larger aortic valve compared to measurements made before CSM.

## Introduction

Left ventricular hypertrophy (LVH) occurs due to primary disease such as hypertrophic obstructive cardiomyopathy (HOCM) or secondary disease, including hypertension and aortic stenosis [1]. Hypertrophic obstructive cardiomyopathy is primarily characterised by asymmetric basal septal hypertrophy (ABSH) where the interventricular septum (IVS) undergoes a greater degree of hypertrophy relative to the remaining of the left ventricle. On the other hand, in secondary disease, a symmetric and uniform pattern of LVH is usually visualised, as an acquired, compensatory mechanism in response to prolonged left ventricular pressure overload [2].

When considering the surgical management of aortic stenosis (AS), the reduction in afterload following aortic valve replacement (AVR) induces a normalisation of left ventricular (LV) haemodynamics including mass regression, reduction in volume and improvement in function [3]. This leads to prognostic and symptomatic benefits for patients. However, 10% of patients with severe AS present with ABSH [1]. The LV undergoes changes mirroring those seen in HOCM and this acts as a site of left ventricular outflow tract obstruction (LVOTO). In the context of AVR for AS, if this ABSH is not corrected during AVR, it is unclear whether it will regress post-operatively and whether the LVOTO will persist. By extension, the risk is that neither the haemodynamically significant LV outflow gradient be abolished nor will diastolic dysfunction resolve. It is likely for this reason that ABSH in patients with AS undergoing AVR has been associated with increased peri-operative morbidity [4]. It is therefore logical to consider addressing ABSH intra-operatively at the time of AVR.

Septal myectomy is the gold standard procedure for symptomatic patients with HOCM refractory to medical therapy with a resting LV outflow gradient ≥ 50 mmHg [5]. Given the common anatomical pathology of ABSH in HOCM and cases of AS, it has been postulated that concomitant septal myectomy (CSM) at the time of AVR for severe AS improves post-operative outcomes [1, 2, 6]

Currently, there is a paucity of evidence to guide the decision-making process regarding the use of AVR with concomitant septal myectomy for severe AS. In a recent review, Magro et al. highlighted that there have been just 5 low evidence retrospective analyses investigating the technique [6]. To our knowledge, most surgeons perform only AVR, which is widely accepted as the gold standard for the treatment of severe or symptomatic AS. The key question is whether AVR with CSM increases the rate of post-operative complications compared to isolated AVR and whether post-operative outcomes are improved.

### Aim

This study aims to present the technique of AVR with CSM for severe AS with ABSH and to determine the early and long-term outcomes regarding morbidity and mortality. Furthermore, this study aims to investigate whether AVR with CSM allows surgeons to increase the size of the prosthetic valve implanted intra-operatively and whether there are any additional advantages elicited from its use.

## Methods

**Fig. 1:**
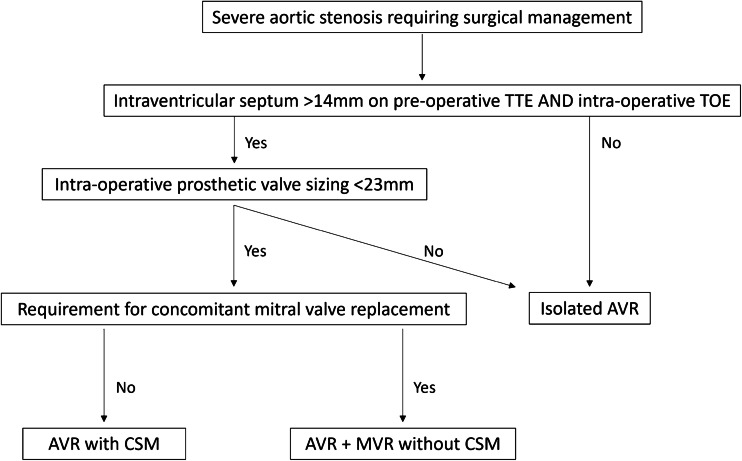
Algorithm outlining the inclusion and exclusion criteria for performing AVR with CSM. AVR, aortic valve replacement; CSM, concomitant septal myectomy; MVR, mitral valve replacement; TOE, transoesophageal echocardiography; TTE, transthoracic echocardiography

Patients were prospectively identified as candidates for AVR with CSM at two centres between 2011 and 2021. The indications for carrying out AVR with CSM are shown in Fig. 1. All patients underwent a transthoracic echocardiogram (TTE) at pre-operative assessment and were considered for CSM if TTE showed severe aortic stenosis with an IVS thickness of over 14 mm. The decision for septal myectomy was made intra-operatively, if the transoesophageal echocardiogram (TOE) showed an IVS thickness > 14 mm and if prosthetic valve sizing was < 23 mm following excision of the diseased aortic valve. Patients were not considered for AVR with CSM if concomitant mitral valve replacement (MVR) was also required. All patients underwent TTE within 7 days post-operatively prior to discharge.

The primary outcomes were operative mortality — defined as death within 30 days of surgery, incidence of post-operative ventricular septal defects (VSD) and prosthetic valve sizing, including index Effective Orifice Area (iEOA). The secondary outcomes were in-hospital complications (stroke or transient ischaemic attack, resternotomy for bleeding, haemofiltration, intra-aortic balloon pump, tracheostomy), permanent pacemaker implantation (PPI), survival at 15 months and post-operative TTE changes within 7 days of discharge, when compared to TTE at pre-assessment.

### Operative technique

**Fig. 2:**
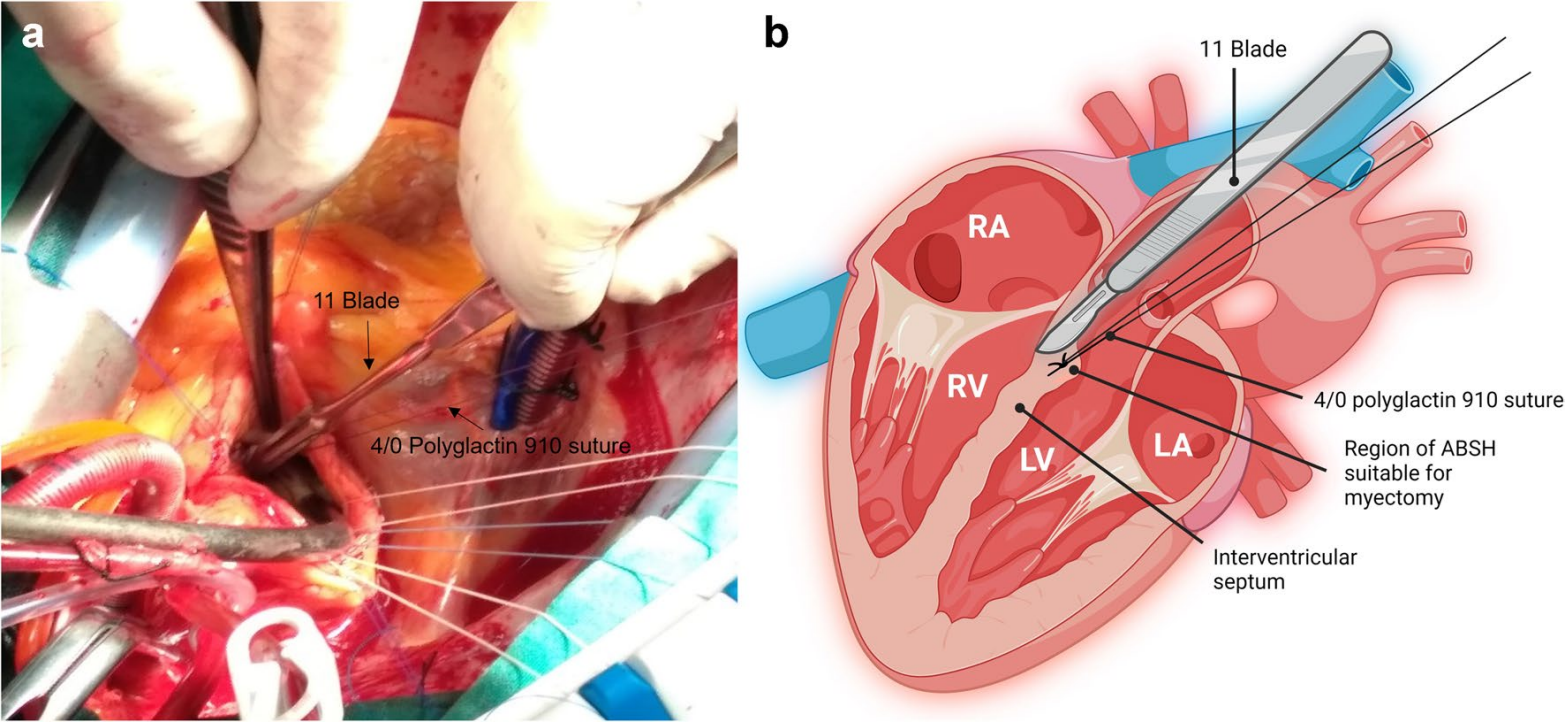
Surgical technique for concomitant septal myectomy. **a** Following excision of the diseased aortic valve, the surgeon pulls on a 4/0 polyglactin 910 suture, to expose the LVOT and anchor the resection of the ventricular septum, which is carried out using an 11 blade. **b** Schematic representation of the technique used to resect hypertrophic septum using 4/0 polyglactin 910 suture and an 11 blade. LA, left atrium; LV left ventricle; LVOT, left ventricular outflow tract, RA; right atrium; RV, right ventricle. Created with BioRender.com

**Fig. 3:**
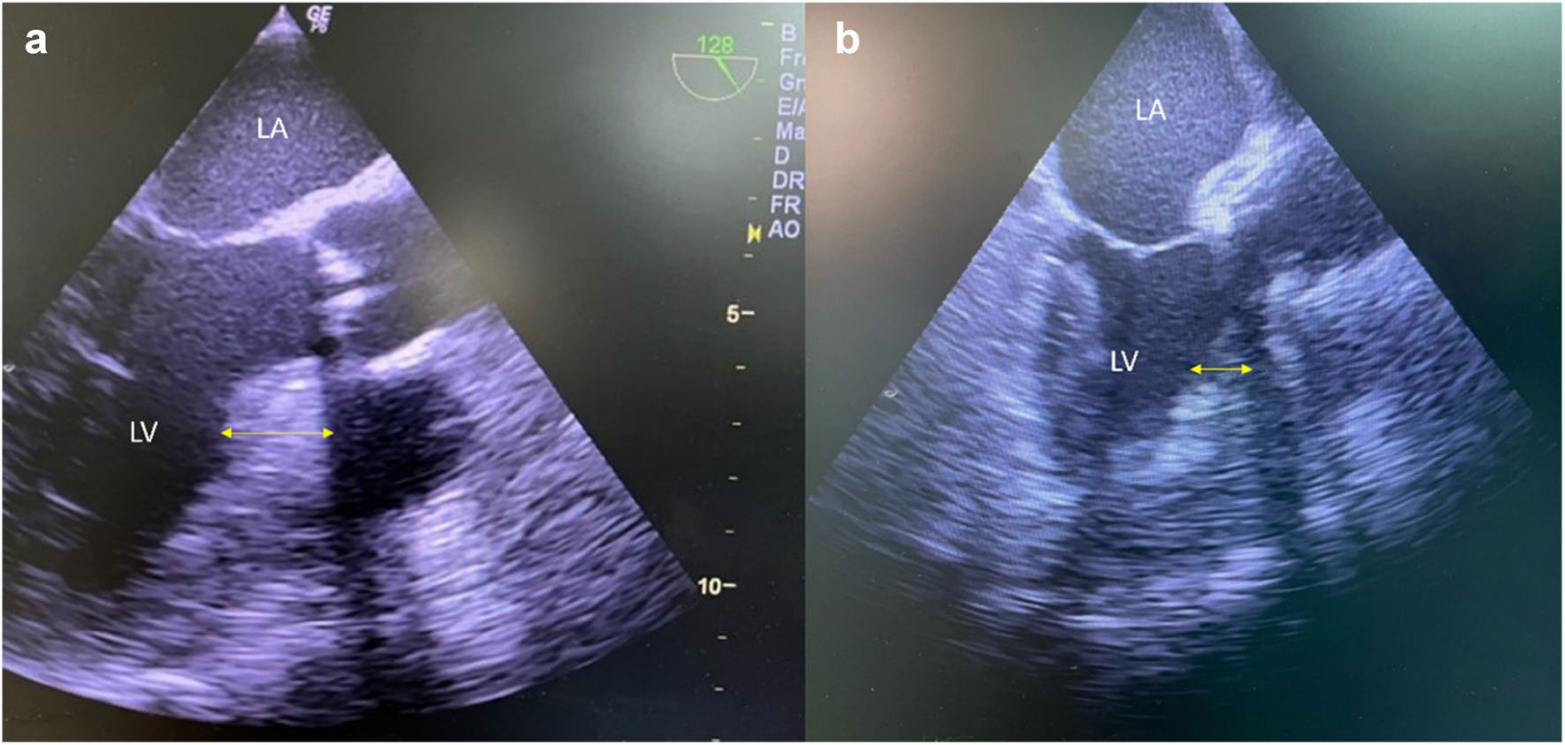
Intra-operative transoesophageal echocardiography **a** before and **b** after concomitant septal myectomy. The yellow arrows highlight the diameter of the interventricular septum, which measures 17 mm in **a** and 7 mm in **b**

Aortic valve replacement was performed with median sternotomy, use of cardiopulmonary bypass and cardioplegic cardiac arrest. Following excision of the diseased aortic valve, but before insertion of the prosthetic valve, CSM was carried out. We followed the operative technique as described by Ralph-Edwards et al. [7]. The operative technique for CSM lasted under 2 min. The right coronary cusp was identified to expose the left ventricular outflow tract (LVOT) inferiorly. A 4/0 polyglactin 910 suture was placed in the centre of the hypertrophic septum to anchor its removal (Fig. 2.). The IVS was resected with an 11 blade with an incision measuring 3 cm vertically by 2 cm horizontally, with a depth of 1 cm. All patients underwent an intra-operative TOE both before and after CSM, to ensure adequate resolution of LVOTO and septal resection (Fig. 3). Additionally, the size of the prosthetic aortic valve was measured both before CSM and after. Following removal of the hypertrophic septum, a prosthetic valve was inserted (either biological or mechanical).

### Follow-up

All patients were followed up in clinic 3 months post-operatively with physical examination, blood tests, electrocardiogram (ECG), chest X-ray and TTE; and 15 months post-operatively with physical examination, ECG and chest X-ray.

### Statistical analysis

Continuous variables are expressed as mean ± standard deviation and categorical variables are expressed as numbers with percentages. Pre-operative and early post-operative measurements on transthoracic echocardiography were compared using Student’s paired *t*-test with a *p* value < 0.05 being statistically significant. Index effective orifice area (iEOA) was calculated with the following calculation: iEOA = Effective orifice area (EOA) / Body surface area (BSA)^2^.

## Results

### Patient demographics

Patient demographics are shown in Table 1. Between 2011 and 2021, fifty-five patients with a diagnosis of severe AS underwent AVR with CSM. Eighteen patients (33%) were female, and thirty-seven patients (67%) were male. The cohort had an average age of 68.3 ± 8.1 years (range 54–80). Mean body surface area was 1.74 ± 0.19 m^2^ (1.41–2.23). Twenty-one patients (38.2%) were hypertensive, sixteen (29.1%) had coronary artery disease, eight had type 2 diabetes mellitus (14.5%), seven had chronic obstructive pulmonary disease (12.7%) and two had carotid stenosis (3.6%). Eight patients (14.5%) had previous myocardial infarction, two patients (3.6%) had previously undergone cardiac surgery, both having undergone MVR, and one patient (1.8%) had a history of stroke.

**Table 1:** Patient demographics. Data reported as mean ± standard deviation (range) for continuous variables and number (%) for categorical variables

Variable	*N* (%) *N* = 55
Age, years (range)	68.3 ± 8.1 (54–80)
Female, sex	18 (32.7)
Male, sex	37 (67.3)
Body surface area, m^2^ (range)	1.74 ± 0.19 (1.41–2.23)
Comorbidities	
Hypertension	21 (38.2)
Diabetes mellitus	8 (14.5)
Chronic obstructive pulmonary disease	7 (12.7)
Coronary artery disease	16 (29.1)
Carotid stenosis	2 (3.6)
History of stroke or transient ischaemic attack	1 (1.8)
History of cardiac surgery	2 (3.6)
History of myocardial infarction	8 (14.5)

### Operative data

Operative data is shown in Table 2. Thirty-two (58.2%) patients underwent only AVR with CSM, whilst in the remaining twenty-three (42.8%) patients, further concomitant procedures were undertaken (fifteen coronary artery bypass grafts (CABG), one CABG and left atrial appendage closure, one ascending aorta replacement, one aortoplasty, one patch pericardial repair of non-coronary sinus of Valsalva, two carotid endarterectomies, two left atrial appendage closures).

**Table 2:** Operative data. Data reported as mean ± standard deviation (range) for continuous variables and number (%) for categorical variables. *AVR*, aortic valve replacement; *CABG*, coronary artery bypass graft; *CSM*, concomitant septal myectomy

Variable	*N* (%) *N* = 55
Cross-clamp time, minutes	59 ± 16.6 (33–115)
Cardiopulmonary bypass time, minutes	81 ± 24.3 (39–178)
Isolated AVR with CSM	32 (58.2)
Concomitant procedures	23 (41.8)
CABG	15 (27.3)
CABG and left atrial appendage closure	1 (1.8)
Ascending aorta replacement	1 (1.8)
Left atrial appendage closure	2 (3.6)
Carotid endarterectomy	2 (3.6)
Patch pericardial repair of non-coronary sinus of Valsalva	1 (1.8)
Aortoplasty	1 (1.8)
Mechanical valves	4 (7.3)
21 mm (intra-annular)	1 (1.8)
23 mm (intra-annular)	1 (1.8)
24 mm (supra-annular)	2 (3.6)
Bioprosthetic valves	51 (92.7)
19 mm	3 (5.5)
21 mm	17 (30.9)
23 mm	31 (56.4)
Indexed effective orifice area	
> 0.85 cm^2^/m^2^	53 (96.4)
≤ 0.85 cm^2^/m^2^	2 (3.6)
≤ 0.75 cm^2^/m^2^	0 (0)
≤ 0.65 cm^2^/m^2^	0 (0)

Four mechanical heart valves were used, of which one was 21 mm, one was 23 mm and two were 24 mm supra-annular valves. Fifty-one bioprosthetic heart valves were used, of which three were 19 mm, seventeen were 21 mm and thirty-one were 23 mm. Valve size to the BSA is shown in Table 3. The iEOA was > 85 cm^2^/m^2^ in 96.4% of cases and ≤ 0.85 cm^2^/m^2^ in the remaining 2 patients (3.6%). No patients had an iEOA ≤ 0.75 cm^2^/m^2^.

**Table 3:** Prosthetic valve size (mm) correlated to body surface area (m^2^) (*n* = 55)

Body surface area (m^2^)										
		1.41–1.50	1.51–1.60	1.61–1.70	1.71–1.80	1.81–1.90	1.91–2.00	2.01–2.10	2.11–2.20	2.21–2.30
Valve size (mm)	19	2	1							
	21		7	6	3	1	1			
	23			4	10	8	6	2	1	1
	24							1	1	

Intra-operatively, at the time of prosthetic valve implantation, it was found in fifty-two cases (94.6%) that the direct valve sizing increased by one size, when compared to size measurement before CSM, without the use of any aortic root enlargement (ARE) techniques. In the remaining three cases, a 19-mm bioprosthetic valve was inserted; however, on measurement prior to CSM, it was not possible to fit this valve size, and were it not for CSM, ARE would have been necessary for these patients.

### Early post-operative outcomes

Early post-operative outcomes are shown in Table 4. One patient died post-operatively (1.8%) following a respiratory tract infection in the intensive care unit (ICU). The remaining fifty-four patients were stepped down from ICU after an average of 1.6 ± 0.4 days (range 1–12) and discharged after an average hospitalisation of 7.15 ± 1.5 days (range 4–20). None of the patients developed a VSD post-operatively. Four patients (7.3%) required pacemaker implantation for complete atrioventricular block. We note that three of these patients had heavily calcified aortic valves, particularly between the right and non-coronary cusps, requiring extensive decalcification. One patient (1.8%) required tracheostomy insertion in ICU. There were no further post-operative complications in this cohort. No patients developed post-operative dynamic LVOTO despite inotropic support.

**Table 4:** Early post-operative outcomes. Data reported as mean ± standard deviation (range) for continuous variables and number (%) for categorical variables. *ICU*, intensive care unit; *VSD*, ventricular septal defect

Outcome	*N* (%) *N* = 55
Mortality (within 30 days)	1 (1.8)
Iatrogenic VSD	0 (0)
Stroke or transient ischaemic attack	0 (0)
Resternotomy for bleeding	0 (0)
Haemofiltration	0 (0)
Intra-aortic balloon pump	0 (0)
Tracheostomy	1 (1.8)
Permanent pacemaker implantation	4 (7.3)
ICU stepdown (days)	1.6 ± 0.4 (1–12)
Hospital discharge (days)	7.15 ± 1.5 (4–20)

### Transthoracic echocardiography

Post-operative changes in TTE, compared to pre-operative TTE are shown in Table 5. The diameter of the interventricular septum was 16.8 ± 1.4 mm (range 14.0–20.4 mm) pre-operatively on TTE and this was significantly reduced (*p* < 0.05) within 7 days post-AVR with CSM to 7.6 ± 1.1 mm (range 4.9–9.8 mm). Furthermore, the diameter of the left ventricular outflow tract on TTE pre-operatively was 20.6 ± 1.2 mm (17.8–22.1 mm) and this was significantly increased (*p* < 0.05) to 23.2 ± 1.4 mm (19.8–25.0 mm). The mean aortic gradient was 55 ± 9.6 mmHg (range 42–85 mmHg) and this was significantly reduced (*p* < 0.05) post-operatively to 17.8 ± 3.5 mmHg (42–85 mmHg).

**Table 5:** Transthoracic echocardiography pre-operatively (within 4 weeks before surgery) compared to early post-operatively (within 7 days after surgery). Data reported as mean ± standard deviation (range)

Variable	Pre-operative	Early post-operative
Interventricular septum (mm)	16.4 ± 1.4 (14.0–20.4)	7.6 ± 1.1 (4.9–9.8)^a^
Left ventricular outflow tract (mm)	20.6 ± 1.2 (17.8–22.1)	23.2 ± 1.4 (19.8–25.0)^a^
Mean aortic gradient (mmHg)	55 ± 9.6 (42–85)	17.8 ± 3.5 (8–32)^a^

^a^*p* < 0.05 pre-operatively compared to early post-operatively

### Long-term outcomes

All fifty-four patients who were successfully discharged following AVR with CSM were followed up in clinic 3 months and 15 months post-operatively. The survival rate was therefore unchanged at 15 months (98.2%). None of the patients required redo operations within this timeframe.

## Discussion

Our aim was to add to the growing, but limited, evidence about the post-operative outcomes of AVR with CSM. In our cohort of fifty-five patients, our results demonstrate that it appears to be a safe procedure when compared to existing literature. The mortality rate of 1.8% does not appear concerning when compared to the known 1–3% rate following AVR [1]. Furthermore, in this cohort, it was secondary to a case of respiratory sepsis which appears to be unrelated to CSM.

Logically, as the septum is being resected in CSM, there is concern about the risk of iatrogenic VSD. Indeed, septal myectomy is a technique that has been designed for HOCM where the degree of hypertrophy is larger than that in AS with ABSH — the septal mass removed in HOCM is 3–12 g, whereas in AS with ABSH this is significantly reduced at approximately 0.8 g [1]. It is therefore harder to demarcate the extent of LVOTO in AS when compared to HOCM and as the demographics include much older patients, the myocardium is less robust and more prone to develop VSD. It is encouraging, therefore, that none of the patients in this study developed a VSD post-operatively, especially when considering that the diameter of the IVS after CSM ranged from 4.9 to 9.8 mm, with a mean of 7.6 mm.

More notable is the 7.3% rate of PPI secondary to complete atrioventricular block which is high when compared to historical 4% incidence following AVR [1]. At both centres at which we carried out this study, the rate of PPI post isolated AVR is < 3%. As mentioned in our results, we noted that three of these four patients had heavily calcified aortic valves, particularly between the right and non-coronary cusps and underwent extensive decalcification intra-operatively. We suspect this to be responsible for the post-operative complete atrioventricular block as opposed to the use of CSM. Nevertheless, we are unable to disregard this figure, especially when considering that in their study, Kayalar et al. also noted similar raised rates of PPI [1]. On the other hand, in their cohort of 29 patients, Di Tommaso et al. reported that no patients required PPI for complete atrioventricular block [2]. To add to this, Lim et al. and Von Aspern et al. recount no significant increase in pacemaker insertion in AVR with CSM compared to isolated AVR [8, 9]. It is also likely our value of 7.3% was due to our small sample size and lack of a control group to compare to; however, we would like to highlight the risk of PPI as something that requires further investigation when considering AVR with CSM.

We hypothesised that the use of CSM would allow for the implantation of a larger valve size when compared to the intra-operative measurement made before CSM, despite the procedure not being an ARE. This is theoretically desirable, as an increased valve size allows for a decreased mean aortic gradient and reduces the risk of patient-prosthesis mismatch (PPM). Indeed, we observed that in fifty-two cases (94.6%) the intra-operative direct valve sizing increased by one size and in the remaining three cases, a size 19-mm bioprosthetic valve was inserted, which, were it not for CSM, would have otherwise required ARE to be appropriately inserted.

It is difficult to know exactly why CSM allows for the insertion of a larger prosthetic valve. However, our theory is that the presence of ABSH mandating CSM decreases the diameter and area of the aortic annulus due to the bulk and stiffness of the hypertrophied interventricular septum underlying the transition point to the aortic root. A smaller aortic annulus is therefore pathological, and following CSM, the annulus regains its compliance and elasticity, such that it can yield to a larger valve size, when inserted. This pathological narrowing of the aortic annulus is also supported by Lim et al. who found that patients who had ABSH requiring CSM, had statistically significant smaller prosthetic valve sizes inserted, when compared to “standard” aortic stenosis patients who did not have ABSH and therefore did not require CSM [8].

Specifically, the conclusion that Lim et al. derived from their series was that “an implanted valve size ≤ 21 mm was the only risk factor for CSM” [8]. This suggests that patients with ABSH requiring CSM are at high risk of PPM, reaffirming our inclusion criteria, as we chose patients who following aortic valve excision had an intra-operative prosthetic valve sizing < 23 mm. This gives patients at the highest risk of PPM and residual high aortic mean and peak gradient an opportunity to have their valve size increased, minimising these complications. Conversely, for patients with valve sizing of ≥ 23 mm, CSM was deemed unnecessary, as they are at low risk of the above complications.

Severe PPM is defined as iEOA < 65 cm^2^/m^2^ and moderate PPM is defined as iEOA < 85 cm^2^/m^2^ [10]. In our cohort, 2 patients (3.6%) had moderate PPM and no patients had severe PPM, whereas in the literature, PPM affects 8–80% of AVR [10]. Our results therefore indicate that CSM in ABSH may be protective against PPM, due to the ability to increase valve size. Multiple studies have shown that the severity of PPM has an inverse relationship to the degree of left ventricular mass regression [10]. Particularly in this cohort of patients who present with ABSH, due to the degree of LVOTO, its persistence due to PPM is arguably catastrophic. This highlights the potential importance of CSM, as it both physically reduces the extent of LVOTO and may reduce the risk of PPM simultaneously.

Interestingly, Kayalar et al., Di Tommaso et al., and Tasca et al. have all shown that CSM provides superior left ventricular remodeling and diastolic function at follow-up [1, 2, 11]. Due to the retrospective nature of each of these studies, none of them commented on the intra-operative decision to be able to increase valve size. It is likely that the superior left ventricular mass regression can be attributed to correction of any LVOTO secondary to ABSH and it is unclear whether PPM prophylaxis played a role. It is important for future research to understand whether this haemodynamic improvement offers patients (1) a decrease in mortality, (2) a decreased incidence of long-term morbidity, and (3) a symptomatic benefit to the patient.

Though our study did not focus on echocardiography, as this has already been covered in the literature, we found that the mean aortic gradient and IVS diameter both decreased significantly and LVOT diameter increased significantly within 7 days post-operatively. These changes were all desired outcomes; however, it is unknown whether they would have occurred progressively without the use of CSM. We believe that the possibility of increasing the size of the prosthetic valve and thereby reversing the potential pathological consequences of ABSH on the aortic annulus outweighs the risks posed by CSM, for the demographic of patient included in this study.

A further issue that needs to be addressed is that there is currently no clear definition of ABSH in the context of AS, as direct visualisation of the IVS is often required to diagnose this. We have already discussed the logic behind utilising an intra-operative prosthetic valve sizing < 23 mm. We also used an IVS diameter of > 14 mm to raise a high clinical suspicion of ABSH, as the 1-cm depth resection in CSM leaves at least 4 mm post-operatively to minimise the risk of iatrogenic VSD. Notably, in isolated AVR, Von Aspern et al. state that there is a trend towards inferior survival when the IVS is > 14 mm, possibly indicating the need for additional intervention such as CSM [9]. We have attempted to identify the patients who would most benefit from AS with ABSH, such that we can move towards a standardised definition that can be used in randomised trials and clinical practice moving forward.

One potential benefit we noticed with CSM is that none of the patients developed post-operative dynamic LVOTO, despite the use of inotropic support, a fact that previous retrospective studies had not investigated [6]. We predict that favourable early post-operative haemodynamic changes may reduce the risk of this, which is important when entering an era of Enhanced Recovery After Surgery, to promote timely stepdown from ICU, early mobilisation and appropriate discharge from hospital [12].

## Study limitations

Limitations of the study are the small population; the short period of patient follow-up which did not extend past 15 months; and the lack of a control group that underwent AVR without CSM.

## Conclusion

Aortic valve replacement with CSM in patients that undergo AVR for symptomatic AS is a simple technique that has shown promising post-operative outcomes, especially regarding no evident increase in mortality, or iatrogenic VSD rate. Most significant is that CSM allows surgeons to increase the size of aortic valve used for patients with ABSH, decreasing the risk of PPM and abolishing the aortic gradient. However, further evidence is required to evaluate whether there is an increased risk of post-operative pacemaker insertion for complete atrioventricular block. A larger-scale randomised trial is warranted to further investigate this operative technique, with particular focus on valve sizing and iEOA.
